# Study on seed-borne cultivable bacterial diversity and antibiotic resistance of *Poa pratensis* L.

**DOI:** 10.3389/fmicb.2024.1347760

**Published:** 2024-01-31

**Authors:** Jinjing Xie, Jie Yang, Shaowei Zhu, Xuan Hou, Haiyan Chen, Xiaoming Bai, Zhenfen Zhang

**Affiliations:** Key Laboratory of Grassland Ecosystem, Ministry of Education, Ministry of Science and Technology, Pratacultural College, Gansu Agricultural University, Lanzhou, China

**Keywords:** commercial variety, wild species, antibiotics, biofilm, swimming motility

## Abstract

In order to study the difference of cultivable seed-borne bacterial diversity between commercial varieties and wild species of *Poa pratensis* L., and their antibiotic resistance to sulfadiazine, tetracycline, oxytetracycline, ciprofloxacin, gentamicin, oxytetracycline and rifampin. In this study, 60 bacterium isolates were isolated by dilution-coated plate method. Through 16S rRNA sequence analysis, 40 representative isolates with different morphological characteristics were identified and phylogenetic tree was constructed. The results of diversity analysis showed that the seed-borne bacterial diversity of commercial varieties was richer than that of wild species. The antibiotic resistance of the isolated bacterial strains was studied by agar dilution method, and it was concluded that the antibiotic resistance of the seed-borne bacteria carried by commercial varieties was stronger than that of the wild species. Finally, the biofilm formation ability and swimming motility of the bacterial strain were measured, and the correlation between the two and the antibiotic resistance of the bacterial strain was analyzed. The analysis showed that the antibiotic resistance of bacterial strains in *Poa pratensis* L. was significantly correlated with their swimming motility. In addition, the swimming motility of the bacterial strains was significantly correlated with the biofilm formation ability. It is worth mentioning that this is the first time to study the drug-resistant bacteria distributed in the seed-borne bacteria of *Poa pratensis* L.

## Graphical abstract

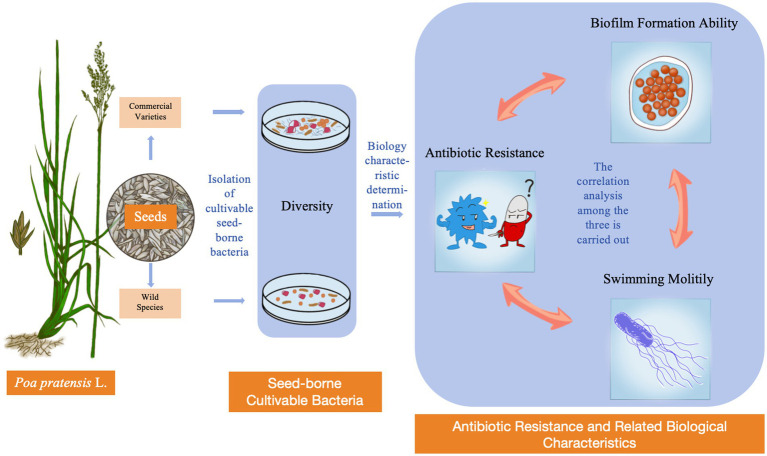

## 1 Introduction

Lawn is the largest ground-covering plant in urban greening, which plays an important role in the process of ecological environment construction. *Poa pratensis* L. is widely cultivated in temperate climate areas because of its high adaptability and high utilization rate in lawn (Gasch et al., 2020). The characteristics of this large area of extensive cultivation have significantly increased the importance of microbial research. This increase in importance is mainly due to the following two reasons. First of all, microorganisms have the characteristics of many kinds, wide distribution, fast reproduction and strong adaptability. Microorganisms can help solve problems related to health, agriculture and the environment. In addition, microorganisms can also spread pathogens and genes (Jogaiah et al., 2016; Satapute et al., 2019). Based on the above characteristics of microorganisms, coupled with the characteristics of wide planting area and large planting area of *Poa pratensis* L., the spread of microorganisms colonized in its tissues has been greatly increased. It is worth mentioning that seeds have special significance as microbial carriers, that is, beneficial seed endophytic bacteria can be selected by plants and transmitted to the next generation through seeds (Truyens et al., 2015). In addition, epiphytic bacteria colonized on the seed surface may be internalized in plant tissues and may also be transmitted vertically or horizontally through commercial, production, and scientific and technological exchanges (Nelson et al., 2018). These epiphytic and endophytic bacteria on seeds are collectively referred to as seed-borne bacteria. Compared with phyllosphere and rhizosphere bacteria, there are few studies in this field in China and abroad.

The composition of the bacterial community in the seed zone is rich and diverse (Cope-Selby et al., 2017; Guo et al., 2021), and the bacteria colonized in different crops, the same crop in different habitats and different varieties of the same crop are different (Normander and Prosser, 2000; Cottyn et al., 2001; Mano and Morisaki, 2008). Based on its rich diversity, its drug resistance has attracted much attention in recent years. This is mainly because since the discovery of antibiotics, it has been continuously used by humans in many fields such as medicine, agriculture, aquaculture and veterinary science. However, antibiotics have poor adsorption in animals. Therefore, most of the antibiotics used in the above fields will be excreted in the form of protoform or metabolites with animal or human feces and urine, and then enter the environment and remain in the environment (Muola et al., 2021; Fuchs et al., 2022). This residual phenomenon is not a good thing, it will have multiple negative effects on animals and plants, ecosystems and even humans. For plants, antibiotics can affect the uptake of nutrients and their regeneration under stress conditions by mainly affecting their roots (Eze et al., 2018; Bai and Cotrufo, 2022).

In addition to causing stress to plants, antibiotics also cause resistance to microorganisms that do not have “intrinsic resistance” colonized on plant tissues. Studies have found that sub-minimal inhibitory concentrations of antimicrobial substances have a significant impact on the physiology and evolution of bacteria. They induce bacterial gene expression, so that some bacteria produce antibiotic resistance (Andersson and Hughes, 2014; Khurshid et al., 2017). The production of these antibiotic resistant bacteria may cause the level of drug resistance genes and potential transmission, which will pose a threat to the health of animals and humans. However, in addition to the risk of transmitting resistance genes, the microorganisms parasitizing on plant tissues can also alleviate the residual situation of antibiotics in the environment by degrading antibiotics in the environment (Yang et al., 2021). This also reduces the production of antibiotic resistant bacteria to a certain extent and reduces the risk of transmission of drug-resistant genes. In summary, it is of great significance to study the resistant microorganisms in plant microorganisms. As an important part of plant microorganisms, are there antibiotic resistant bacteria in seed-borne bacteria? At present, there are few studies on this aspect in China and abroad.

In recent years, the research on bacterial resistance has focused on its mechanism. Among these mechanisms studied, biofilm formation is a particularly important part. Biofilms have a high degree of structure and strong adhesion. Once formed, they can provide mechanical stability and resistance to environmental stress for bacteria, which makes it extremely difficult to kill embedded bacteria (Zheng et al., 2021). This property can be seen as a biological barrier that limits the amount of antimicrobial agents entering the cell, reduces the effective concentration of antimicrobial agents, and causes bacteria to develop resistance (Capita and Alonso-Calleja, 2013). In the process of biofilm formation, the motility of bacteria plays an important role. The surface attachments (such as flagella) of motile bacteria can play an important role in the adhesion process by inducing motile bacteria to produce a faster dynamic response to surface properties. After bacteria adhere to the host surface, motile bacteria attract free bacteria through chemotaxis and quorum sensing, thus forming biofilm faster than non-motile bacteria (Gutman et al., 2013). In addition, motility also gives cells the selective advantage of escaping from harsh conditions and finding a more favorable environment. This feature can reduce the exposure of bacteria to antimicrobial drugs, thereby reducing the damage to bacteria and making bacteria resistant (Koskella et al., 2011; Samad et al., 2017). Therefore, it is necessary to study the biofilm formation ability and motility of bacteria for the study of antibiotic resistance.

In this study, the seed-borne bacteria of commercial varieties and wild species of *Poa pratensis* L. were isolated and identified, and the sensitivity of each bacterial strain to different antibiotics was studied. The biofilm formation ability and motility were studied, and the relationship between the two and bacterial resistance was discussed. In this study, the diversity of culturable bacteria in *Poa pratensis* L. was clarified, and the distribution of drug-resistant bacteria in *Poa pratensis* L. was preliminarily investigated, which laid a theoretical and practical foundation for the study of the transmission range and transmission route of drug-resistant bacteria or drug-resistant genes between plants and the environment.

## 2 Materials and methods

### 2.1 Experimental materials

The tested seeds of *Poa pratensis* L. were provided by the Laboratory of Forage Germplasm Resources of Gansu Agricultural University (Table 1).

**Table 1:** Seed information of *Poa pratensis* L.

Variety	Seed source	Storage life
“Midnight” *Poa pratensis* L.	CLOVER Eco-Technology Co., Ltd. (Origin: United States)	1
“Lucky Goddess” *Poa pratensis* L.	CLOVER Eco-Technology Co., Ltd. (Origin: United States)	1
“Black Jack” *Poa pratensis* L.	CLOVER Eco-Technology Co., Ltd. (Origin: United States)	1
Anding wild *Poa pratensis* L.	Origin: Anding District, Dingxi City, Gansu Province	2
Yuzhong wild *Poa pratensis* L.	Origin: Yuzhong County, Lanzhou City, Gansu Province	2
Weiyuan wild *Poa pratensis* L.	Origin: Weiyuan County, Dingxi City, Gansu Province	2

The test medium included Casein Soya Bean Digest Agar (TSA), for the isolation and purification of cultivable seed-borne bacteria and determination of antibiotic resistance; Tryptone Soy Broth (TSB), for bacterial culture in biofilm formation assay (Viksne et al., 2023); Swimming medium, for the determination of bacterial swimming motility (Qu et al., 2014). The tested antibiotics: Sulfadiazine, tetracycline, oxytetracycline, ciprofloxacin, rifampin, ceftazidime and gentamicin. The above antibiotics were purchased from Beijing Solarbio Technology Co., Ltd.

### 2.2 Experimental design

#### 2.2.1 Configuration of test medium

Casein Soya Bean Digest Agar (TSA): Tryptone or casein peptone pancreatic digest 17.0 g, soybean tryptone 3.0 g, NaCl 5.0 g, K_2_HPO_4_ 2.5 g, gluconate 2.5 g, agar 20.0 g, distilled water 1,000 mL, PH7.3, sterilization at 121°C for 20 min, then pour into the culture dish.

Tryptone Soy Broth (TSB): Tryptone or casein peptone pancreatic digest 17.0 g, soybean tryptone 3.0 g, NaCl 5.0 g, K2HPO4 2.5 g, gluconate 2.5 g, distilled water 1,000 mL, PH7.3, sterilization at 121°C for 20 min.

Swimming medium: Beef extract 3.0 g, proteose peptone 10.0 g, NaCl 5.0 g, agar 3.0 g, distilled water 1,000 mL, pH 7.0 ~ 7.2, sterilized at 121°C for 20 min, poured into the medium.

#### 2.2.2 Isolation of cultivable seed-borne bacteria

Isolation of cultivable seed-borne bacteria in *Poa pratensis* L. was carried out by dilution coating plate method (Zhang, 2013).

First, 0.5 g of seeds were weighed and placed in 1% NaClO for disinfection for 5 min, and the soaked seeds were rinsed with sterile distilled water for many times until the pungent odor of NaClO disappeared. The washed seeds were placed in a sterile mortar, and 10 mL of sterile water was added several times for grinding. After fully grinding and standing for more than 5 min, the clear supernatant was the original bacterial suspension. Set sterile distilled water as the control group.

Then, 100 μL of the supernatant obtained after the mortar was placed was uniformly diluted to 900 μL of sterile water to obtain a 10-fold dilution. Then, 100 μL of the supernatant obtained after standing in the mortar was uniformly diluted to 900 μL of sterile water to obtain a 10-fold dilution. Diluted by 10 times in turn, the diluents of 10^−1^, 10^−2^, 10^−3^, 10^−4^, 10^−5^, and 10^−6^ were obtained.

Subsequently, 100 μL of each gradient dilution was transferred to the center of the TSA with a pipette gun (20 ~ 200 μL), and evenly applied to the entire medium with a applicator, with 3 replicates in each treatment. Then 100 μL of each gradient dilution was transferred to the center of TSA with a pipettor (20 ~ 200 μL), and evenly applied to the whole medium with a spreader, with 3 replicates in each treatment. The culture dishes inoculated with bacteria and sterile distilled water were placed in a constant temperature incubator at 28 ± 1°C for 72 h in the dark to observe the results. After confirming that the growth of bacteria was in good condition, the observation and statistics were carried out.

Finally, the morphological characteristics of the colonies were observed, and the total number of colonies with single colonies number between 30 and 300 and no spreading growth was counted. The purified bacteria were stored in LB supplemented with glycerol at −80°C for a long time.

#### 2.2.3 Determination of antibiotic resistance of cultivable seed-borne bacteria

The agar dilution method was used to determine the cultivable seed-borne bacterial antibiotic resistance (Jiang et al., 2022; Tian et al., 2023).

Different concentrations of antibiotics were added to the agar medium to configure the medium for the determination of antibiotic resistance. The concentrations of each antibiotic were set to 1, 3, 5, 10, 20, 40, 80, 160, 320, 640, 1,280, 2,560 mg L ^−1^. Subsequently, the isolated bacteria were inoculated into TSB and cultured in a constant temperature incubator at 28 ± 1°C for 24 h in darkness. After the end of culture, the bacterial suspension was diluted so that the optical density of the bacterial suspension at 600 nm wavelength was in the range of 0.25–0.3. Then, 2 μL of bacterial suspension was inoculated on the surface of agar medium containing different antibiotic concentrations, and each treatment was repeated 5 times. After 48 h of culture at 37°C ± 1°C, the growth of bacteria was observed. Finally, the minimum inhibitory concentration (MIC) of the inoculum was read. MIC is the lowest antibiotic concentration that completely inhibits visible growth.

#### 2.2.4 Determination of biofilm formation ability of cultivable seed-borne bacteria

The ability of bacterial biofilm formation was determined by crystal violet staining (Viksne et al., 2023).

Single colonies formed on TSA were inoculated into TSB and cultured at 37°C for 16–18 h. Then, 150 μL bacterial solution was inoculated into a sterile 96-well plate. Each 96-well plate contained 22 bacterial strains, the first was listed as a negative control (uninoculated medium), and each experiment was repeated three times. Subsequently, the inoculated 96-well plates were incubated at 37°C for 48 h.

Staining experiments were performed after 48 h of incubation. Firstly, the bacterial liquid in the 96-well plate was gently poured out, and all the holes were emptied. Wash each well with 250 μL 0.9% sterile saline. After washing 3 times, 150 μL 0.1% crystal violet was added to each hole for dyeing. After standing for 15 min, the crystal violet dye solution in the hole was gently removed, and 250 μL of distilled water was added to each hole for washing. After washing 3 times, 150 μL 96% ethanol was added to each well. Finally, the optical density (OD) of the hole was measured at a wavelength of 570 nm using a microplate spectrophotometer.

#### 2.2.5 Determination of swimming motility of cultivable seed-borne bacteria

The single colonies on TSA were picked with sterile toothpicks and inoculated on the swimming medium by puncture method, and cultured at 28°C for 20 h. At the 20th hour, the diameter of the turbid area formed by the migration of bacteria from the inoculation point was observed and measured, and the test results were recorded (Qu et al., 2014).

### 2.3 Measurement of relevant indicators

#### 2.3.1 Plate colony count of cultivable seed-borne bacteria

Plate colony count of cultivable seed-borne bacteria: Number of bacteria (CFU · g ^−1^) = 10 MD/W, Where, M represents the average total number of colonies per dish, D represents the dilution factor, W represents the quality of plant tissue (g).

#### 2.3.2 Single colony 16S rRNA sequencing of cultivable seed-borne bacteria

Single colonies were selected for purified culture, and bacterial genomic DNA extraction kit (purchased from Tiangen Biochemical Technology Co., Ltd.) was used to extract bacterial DNA and perform PCR amplification. The amplified products were sent to Shanghai Personal Biotechnology Co., Ltd. for sequencing.

#### 2.3.3 Construction of phylogenetic tree of cultivable seed-borne bacteria

The sequences obtained by sequencing were submitted to NCBI-Blast for online comparison. Five strains with high homology were selected as reference objects, and the phylogenetic tree was constructed by using the Neighbor-joining (NL) method in MEGA 11 software.

#### 2.3.4 Judgment of the ability of biofilm formation of cultivable seed-borne bacteria

By comparing the average OD value of each well measured at the wavelength of 570 nm, the average value was compared with the size of ODC, 2ODC and 4ODC to divide the biofilm formation ability of the strain. ODC was defined as the average OD value of the negative control. The division of biofilm formation ability of bacterial strains is as follows: OD ≤ ODC = No biofilm formation ability, ODC < OD ≤ 2ODC = Weak biofilm formation ability, 2ODC < OD ≤ 4ODC = Medium biofilm formation ability, OD ≥ 4ODC = Strong biofilm formation ability (Stepanović et al., 2007).

### 2.4 Statistical analysis

All the measured data in the experiment were sorted out using Excel 2021; Data were evaluated with variance analysis using SPSS 27.0. The data were expressed as “average mean ± standard deviation”, and the difference was statistically significant when the statistical test level *p* < 0.05. Correlation analysis and mapping were performed using the online software Chiplot.^1^

## 3 Results

### 3.1 Phenotypic characteristics of cultivable seed-borne bacterium isolates

**Figure 1:**
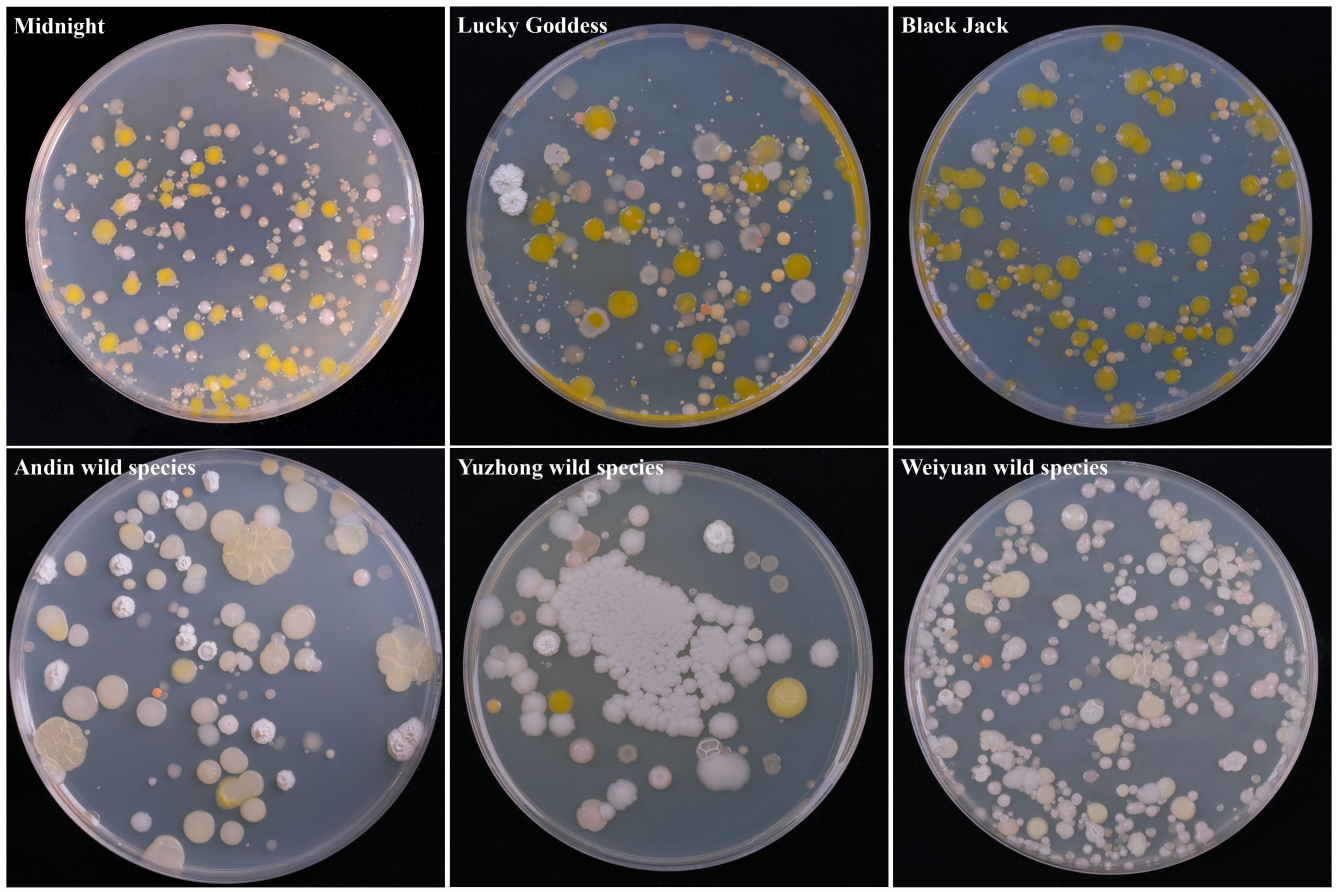
Morphological characteristic of cultivable bacterium isolates from seed of *Poa pratensis* L.

A total of 60 bacterium isolates were isolated from 6 *Poa pratensis* L. seed samples, and their morphological characteristics are shown in Figure 1. It can be seen from the figure that the bacterium isolates isolated from the seeds of *Poa pratensis* L. have great differences in morphology.

### 3.2 Phylogenetic tree of cultivable seed-borne bacteria

**Figure 2:**
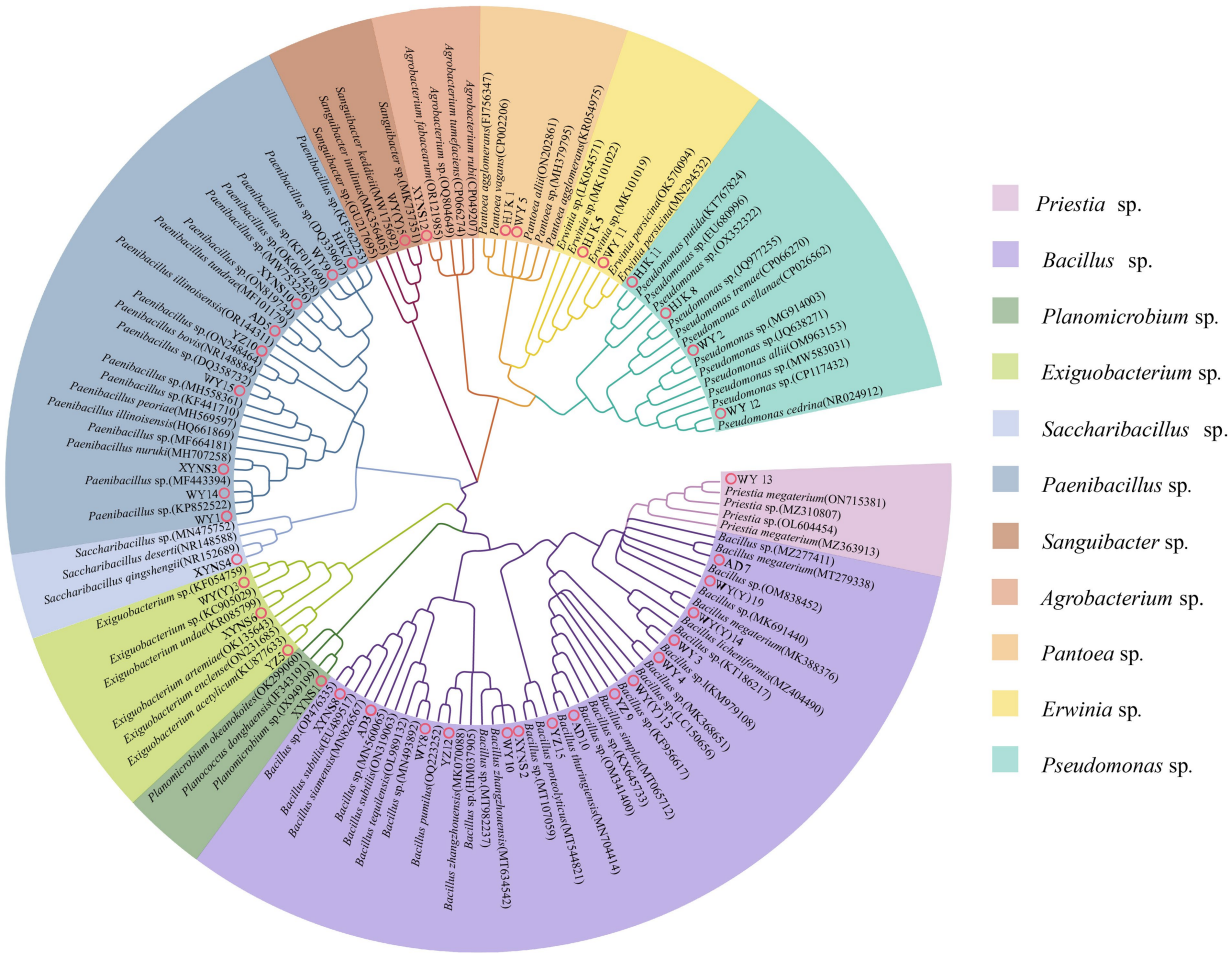
16S rRNA phylogenetic tree of cultivable seed-borne bacteria in *Poa pratensis* L.

16S rRNA sequencing and phylogenetic tree construction were performed on 40 representative bacterium isolates with the different morphological characteristics, and the results were shown in Figure 2. The results showed that a total of 11 genera of bacteria were isolated and identified from the seeds of *Poa pratensis* L. The results of phylogenetic tree construction showed that WY3, WY4, WY8, WY10, XYNS2, XYNS8, YZ9, YZ12, YZ15, AD3, AD7, AD10, WY (Y) 14, WY (Y) 15, and WY (Y) 19 were clustered with *Bacillus* spp.; WY1, WY9, WY14, WY15, XYNS3, XYNS10, HJK7, YZ10, and AD5 were clustered with *PaeniBacillus* spp.; WY2, WY12, HJK8, and HJK11 were clustered with *Pseudomonas* spp.; XYNS6, YZ5 and WY (Y) 3 were clustered with *Exiguobacterium* spp.; WY5 and HJK1 were clustered with *Pantoea* spp.; WY11 and HJK5 were clustered with *Erwinia* spp.; WY13 was clustered with *Priestia* sp.; WY (Y) 5 was clustered with *Sanguibacter* sp.; XYNS12 was clustered with *Agrobacterium* sp.; XYNS4 was clustered with *Saccharibacillus* sp.; XYNS1 was clustered with *Planomicrobium* sp. The accession number of the identified bacterial strains is shown in Table 2.

**Table 2:** Cultivable species of *Poa pratensis* L. with bacterial genus level classification and accession number of bacterial strains.

Code	Number/(CFU·g^−1^)	Scientific name	Accession
WY1	6 × 10^3^	*PaeniBacillus* sp.	OR857423
WY2	6 × 10^3^	*Pseudomonas* sp.	OR857426
WY3	6 × 10^3^	*Bacillus* sp.	OR857430
WY4	4 × 10^3^	*Bacillus* sp.	OR857432
WY5	4.8 × 10^4^	*Pantoea* sp.	OR857462
WY8	2 × 10^3^	*Bacillus* sp.	OR857442
WY9	2.6 × 10^4^	*PaeniBacillus* sp.	OR857445
WY10	4 × 10^3^	*Bacillus* sp.	OR857448
WY11	4 × 10^3^	*Erwinia* sp.	OR857451
WY12	6 × 10^3^	*Pseudomonas* sp.	OR857452
WY13	2 × 10^3^	*Priestia* sp.	OR857455
WY14	8 × 10^3^	*PaeniBacillus* sp.	OR857456
WY15	2 × 10^3^	*PaeniBacillus* sp.	OR857459
XYNS1	2 × 10^3^	*Planomicrobium* sp.	OR857425
XYNS2	2 × 10^3^	*Bacillus* sp.	OR857427
XYNS3	1 × 10^4^	*PaeniBacillus* sp.	OR857431
XYNS4	1.6 × 10^4^	*SacchariBacillus* sp.	OR857433
XYNS6	1.4 × 10^4^	*Exiguobacterium* sp.	OR857438
XYNS8	4 × 10^3^	*Bacillus* sp.	OR857443
XYNS10	1.2 × 10^4^	*PaeniBacillus* sp.	OR857449
XYNS12	4 × 10^3^	*Agrobacterium* sp.	OR857453
HJK1	1.26 × 10^5^	*Pantoea* sp.	OR857424
HJK5	2 × 10^3^	*Erwinia* sp.	OR857437
HJK7	2 × 10^3^	*PaeniBacillus* sp.	OR857440
HJK8	1 × 10^3^	*Pseudomonas* sp.	OR857441
HJK11	4 × 10^3^	*Pseudomonas* sp.	OR857450
YZ5	6 × 10^3^	*Exiguobacterium* sp.	OR857436
YZ9	8 × 10^3^	*Bacillus* sp.	OR857444
YZ10	2 × 10^4^	*PaeniBacillus* sp.	OR857446
YZ12	1.4 × 10^4^	*Bacillus* sp.	OR857454
YZ15	2.2 × 10^5^	*Bacillus* sp.	OR857458
AD3	4 × 10^4^	*Bacillus* sp.	OR857428
AD5	1.2 × 10^4^	*PaeniBacillus* sp.	OR857434
AD7	6 × 10^3^	*Bacillus* sp.	OR857439
AD10	2 × 10^3^	*Bacillus* sp.	OR857447
WY(Y)3	2 × 10^3^	*Exiguobacterium* sp.	OR857429
WY(Y)5	6 × 10^3^	*Sanguibacter* sp.	OR857435
WY(Y)14	1.6 × 10^4^	*Bacillus* sp.	OR857457
WY(Y)15	1 × 10^4^	*Bacillus* sp.	OR857460
WY(Y)19	1 × 10^4^	*Bacillus* sp.	OR857461

### 3.3 Community analysis of the mix-culture in cultivable seed-borne bacteria

**Figure 3:**
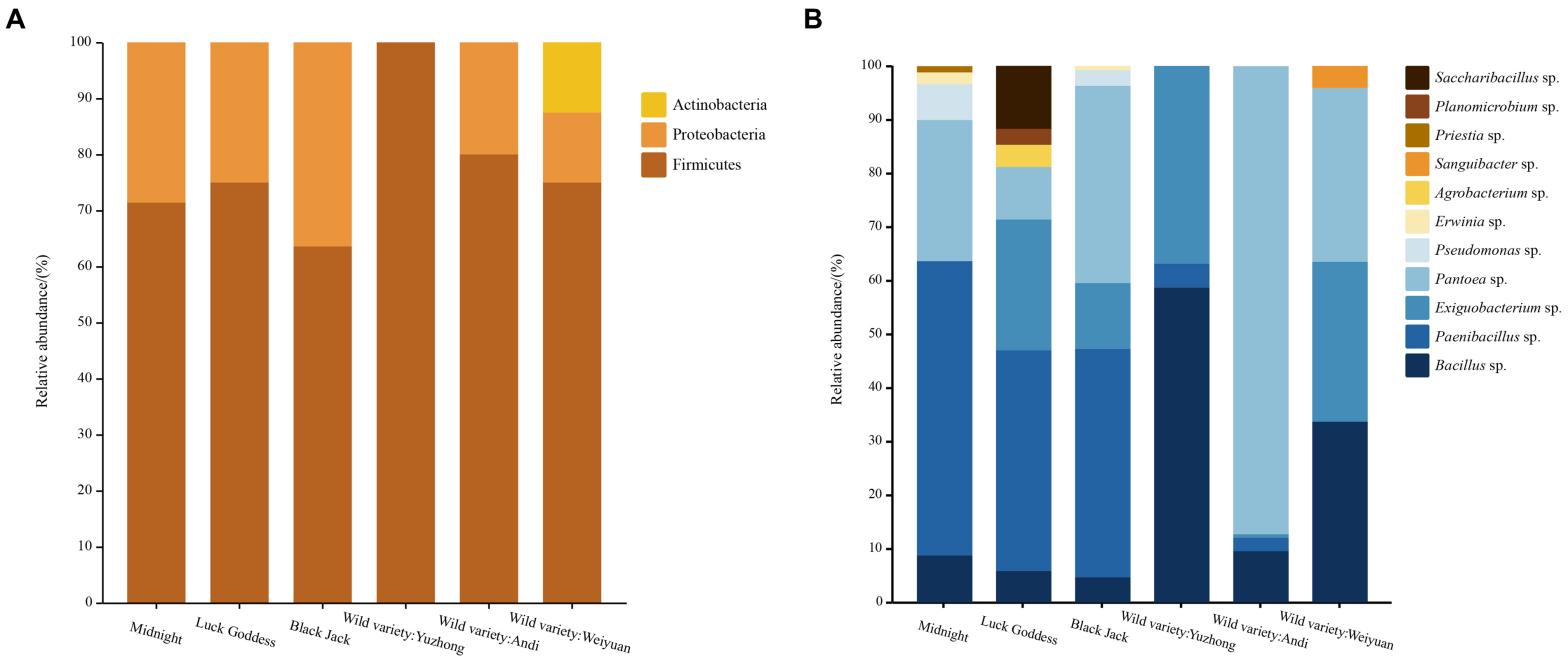
Distribution of cultivable seed-borne bacteria in *Poa pratensis* L. at family **(A)** and genus **(B)** level.

From the level of phylum classification, the seed-borne bacteria in *Poa pratensis* L. were mainly from Firmicutes, Proteobacteria, and Actinobacteria, as shown in Figure 3. In addition to the wild *Poa pratensis* L. from Anding, the other varieties of bacteria were dominated by Firmicutes, and the relative abundance in these samples was more than 60%. The first dominant phylum of wild *Poa pratensis* L. from Anding was Proteobacteria, and the relative abundance reached 87.5%. The second dominant phylum with bacteria was Proteobacteria in all varieties except wild *Poa pratensis* L. form Anding. The relative abundance of Proteobacteria in the seeds of “Midnight”, “Lucky Goddess”, “Black Jack”, and Weiyuan wild *Poa pratensis* was 35.26, 13.72, 40.34, and 32.47%, respectively. Actinobacteria was only isolated from the seeds of Weiyuan wild *Poa pratensis* L., and its relative abundance was only 12.5%.

A total of 11 genera of bacteria in each sample were isolated from the genus level. They are *Bacillus* spp., *PaeniBacillus* spp., *Exiguobacterium* spp., *Pantoea* spp., *Pseudomonas* spp., *Erwinia* spp., *Agrobacterium* sp., *Sanguibacter* sp., *Priestia* sp., *Planomicrobium* sp., and *SacchariBacillus* sp., respectively. The results are shown in Figure 2. Among the six tested samples, the “Lucky Goddess” seed-borne bacteria had the most abundant diversity at the genus level, including 7 genera, of which *PaeniBacillus* spp. was the first dominant genus, with a relative abundance of 41.18%. The diversity of bacteria in the wild *Poa pratensis* L. species from Yuzhong was relatively scarce at the genus level, with only three genera, of which *Bacillus* spp. also was the main genus, with a relative abundance of 58.84%. Among the three commercial *Poa pratensis* L. varieties, *PaeniBacillus* spp. was the first dominant genus, and the relative abundance was more than 40%. Among the three wild species, except for the wild *Poa pratensis* L. from Yuzhong, the *Pantoea* spp. was dominant in the seed-borne bacteria of other two wild species. Especially in the bacteria of the wild *Poa pratensis* L. from Anding, the relative abundance of *Pantoea* spp. reached 87.5%.

### 3.4 Community structure analysis in cultivable seed-borne bacteria

**Figure 4:**
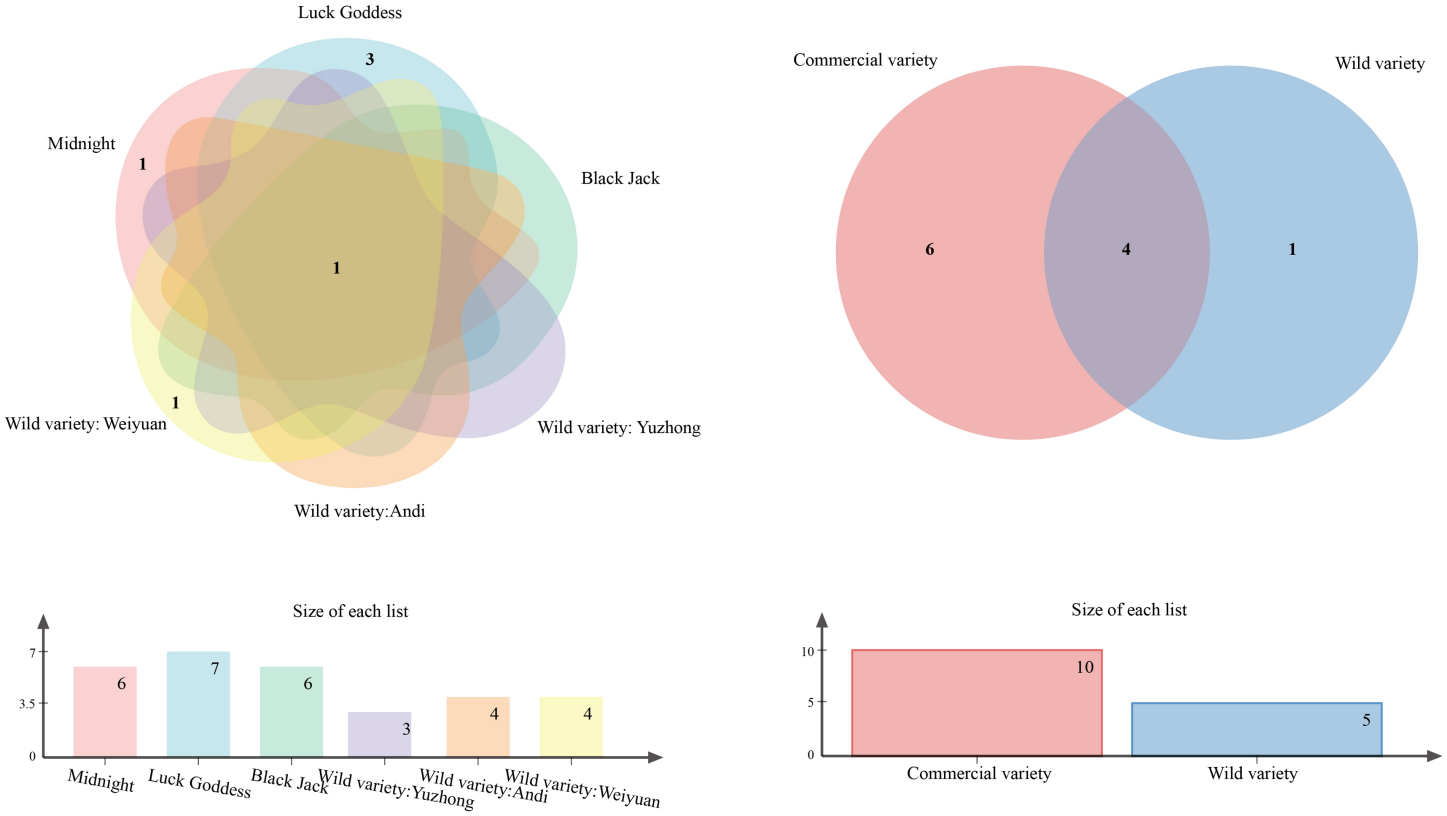
Venn diagram of cultivable seed-borne bacteria in *Poa pratensis* L. at genus level.

At the genus classification level, the Venn diagram shows the common and unique genera of the seed-borne bacteria of each sample, as shown in Figure 4. The results showed that there was one common genus in the six samples of *Poa pratensis* L., which was *Bacillus* sp. Among them, there are three unique genera in the species of “Lucky Goddess”, which are *Agrobacterium* sp., *Planomicrobium* sp. and *Saccharibacillus* sp. There were one unique genus in the bacteria of “Midnight”and Weiyuan wild *Poa pratensis* L., which were *Priestia* sp. and *Sanguibacter* sp., respectively. This indicates that among all samples, seed-borne bacteria in “Lucky Goddess” *Poa pratensis* L. have the highest specificity and the most complex community structure; the specificity of bacteria in “Black Jack” *Poa pratensis* L, Anding and Yuzhong wild *Poa pratensis* L. was relatively low, and the community structure was single. Venn diagram was used to analyze the bacterial community structure of wild and commercial varieties. The results showed that there were 4 common genera between them, which were *Bacillus* spp., *PaeniBacillus* spp., *Exiguobacterium* spp. and *Pantoea* spp. Between the two, there are 6 unique genera of seed-borne bacteria in commercial varieties, and only 1 unique genus in wild species. The results showed that compared with wild species, commercial varieties had higher specificity and more complex community structure.

### 3.5 Antibiotic resistance of cultivable seed-borne bacteria

**Figure 5:**
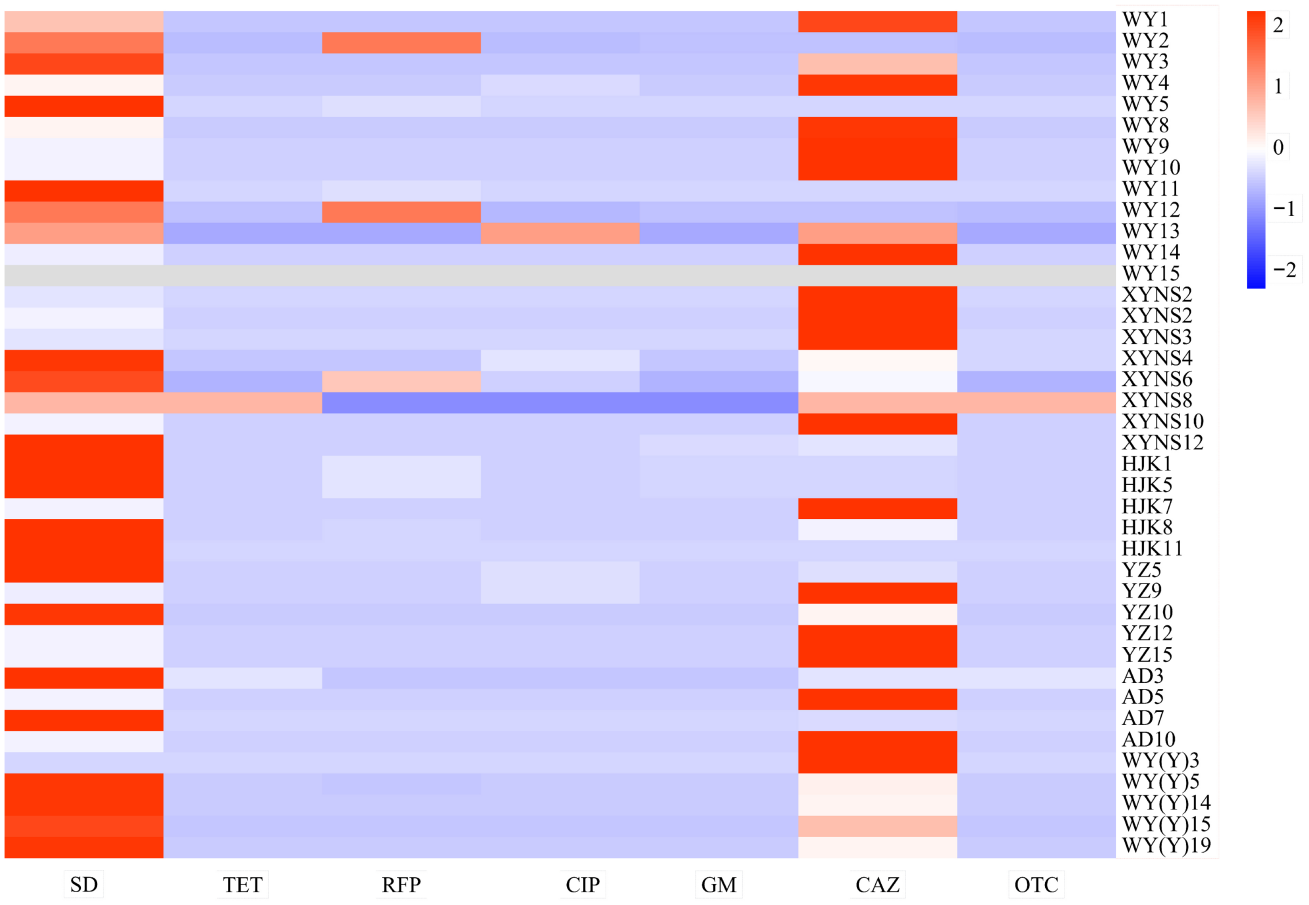
Heatmap of drug resistance distribution of cultivable seed-borne bacteria in *Poa pratensis* L.

The results showed that the resistance of different bacterial strains of *Poa pratensis* L. to different kinds of antibiotics showed great differences. In addition, the antibacterial ability of sulfadiazine and ceftazidime against the isolated seed-borne bacteria was weaker than that of other types of antibiotics, so they show higher minimum inhibitory concentration, as shown in Figure 5.

It can be seen from Table 3 that sulfadiazine showed the weakest antibacterial ability to the isolated strains among the tested antibiotics. More than 90% of the bacterial strains could grow normally on the agar medium containing 1 mg · L ^−1^ sulfadiazine, and the bacteria with the strongest resistance to sulfadiazine could completely inhibit the visible growth of bacteria when the concentration of sulfadiazine reached 2,560 mg · L ^−1^. Rifampicin, oxytetracycline, ciprofloxacin, tetracycline and gentamicin showed comparable antibacterial ability to the isolated bacterial strains. Among the isolated bacterial strains, only less than 35% of the bacterial strains grew at the above antibiotic concentration of 1 mg · L ^−1^, and when the concentration of tetracycline and oxytetracycline was 5 mg · L ^−1^, the visible growth of all isolated strains could be completely inhibited. In summary, tetracycline, rifampicin, ciprofloxacin, gentamicin and oxytetracycline have stronger antibacterial ability and wider antibacterial range against seed-borne bacteria in *Poa pratensis* L.

**Table 3:** The growth of cultivable seed-borne bacteria of *Poa pratensis* L. on media containing different concentrations of antibiotics.

%	SD		TET		RFP		CIP		GM		CAZ		OTC	
	C	W	C	W	C	W	C	W	C	W	C	W	C	W
1 mg·L^−1^	96.15	92.86	30.77	14.29	34.62	0	26.92	21.43	30.77	14.29	96.15	100.00	34.62	14.29
3 mg·L^−1^	96.15	92.86	15.38	0	26.92	0	26.92	14.29	26.92	0	84.62	64.29	3.85	0
5 mg·L^−1^	96.15	92.86	0	0	26.92	0	7.69	0	7.69	0	76.92	50.00	0	0
10 mg·L^−1^	69.23	64.29	0	0	26.92	0	0	0	0	0	61.54	50.00	0	0
20 mg·L^−1^	69.23	50.00	0	0	23.08	0	0	0	0	0	30.77	42.85	0	0
40 mg·L^−1^	53.85	42.86	0	0	23.08	0	0	0	0	0	26.92	35.71	0	0
80 mg·L^−1^	26.92	7.14	0	0	0	0	0	0	0	0	26.92	21.43	0	0
160 mg·L^−1^	23.08	0	0	0	0	0	0	0	0	0	23.08	21.43	0	0
320 mg·L^−1^	19.23	0	0	0	0	0	0	0	0	0	23.08	21.43	0	0
640 mg·L^−1^	19.23	0	0	0	0	0	0	0	0	0	0	0	0	0
1,280 mg·L^−1^	7.69	0	0	0	0	0	0	0	0	0	0	0	0	0
2,560 mg·L^−1^	0	0	0	0	0	0	0	0	0	0	0	0	0	0

In the table, C denotes commercial species and W denotes wild species.

After comparing the antibiotic resistance of cultivable seed-borne bacteria in commercial varieties and wild species of *Poa pratensis* L., it was found that the antibiotic resistance of strains isolated from commercial varieties of *Poa pratensis* L. seeds was stronger than that of wild species. As shown in Table 3, the highest concentration of commercial varieties-borne bacteria that can grow on other tested antibiotics except ceftazidime is higher than that of wild species-borne bacteria. Although the highest concentration of ceftazidime for the normal visible growth of bacteria in commercial and wild species was consistent, the percentage of bacterial strains in commercial varieties that could grow on the agar medium containing ceftazidime at this concentration was higher than that of wild species. In summary, the resistance of bacteria isolated from the commercial varieties of *Poa pratensis* L. to antibiotics was stronger than that of wild species.

### 3.6 Biofilm-forming ability of seed-borne bacteria

**Figure 6:**
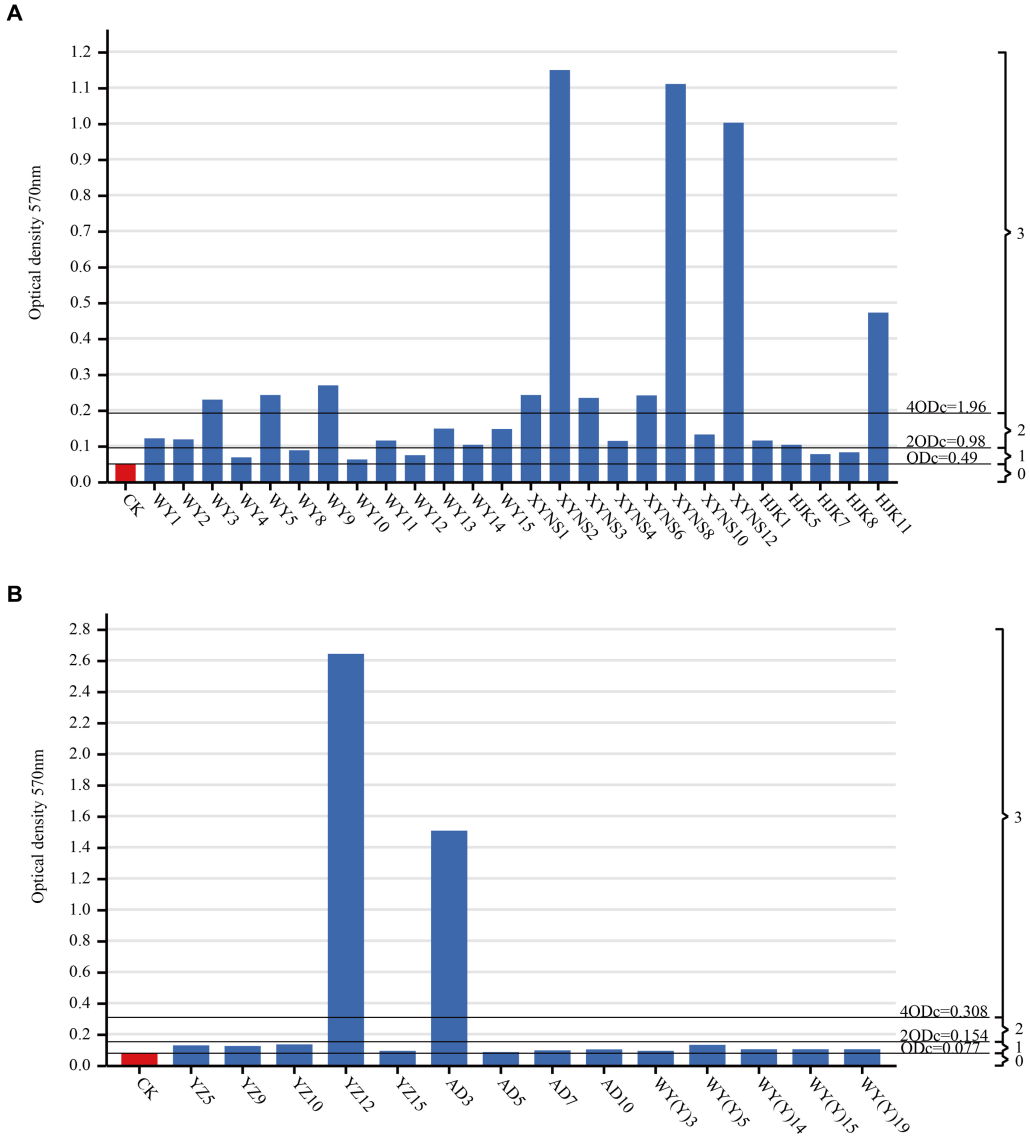
Biofim formation capability on microtiter plate of cultivable seed-borne bacteria in *Poa pratensis* L., commercial varieties **(A)**, wild species **(B)**. CK as a negative control. ODc and biofilm formation ability were marked with a horizontal line: 0-biofilm nonproducers; 1-weak biofilm producers; 2-medium biofilm producers; 3-strong biofilm producers.

By measuring the biofilm formation ability of cultivable seed-borne bacteria in *Poa pratensis* L., it was found that among the three commercial varieties, the number of strains with strong and medium biofilm formation ability was the same, 10 bacterial strains each, accounting for 38.46% of the bacterial strains isolated from commercial products. There were only 6 bacterial strains with weak biofilm formation ability, accounting for 23.06% of the bacterial strains isolated from commercial varieties. Among the three wild species, the strains with weak biofilm formation ability were dominant, with a total of 12 bacterial strains, accounting for 85.71% of the isolated wild species with bacterial strains; only 2 bacterial strains had strong biofilm formation ability, accounting for 14.29%; there was no bacterial strain with moderate biofilm formation ability, and the results are shown in Figure 6. Therefore, it can be seen that the biofilm formation ability of commercial varieties is generally stronger than that of wild species in all seed-borne pathogens isolated from *Poa pratensis* L.

### 3.7 Swimming motility of cultivable seed-borne bacteria

**Figure 7:**
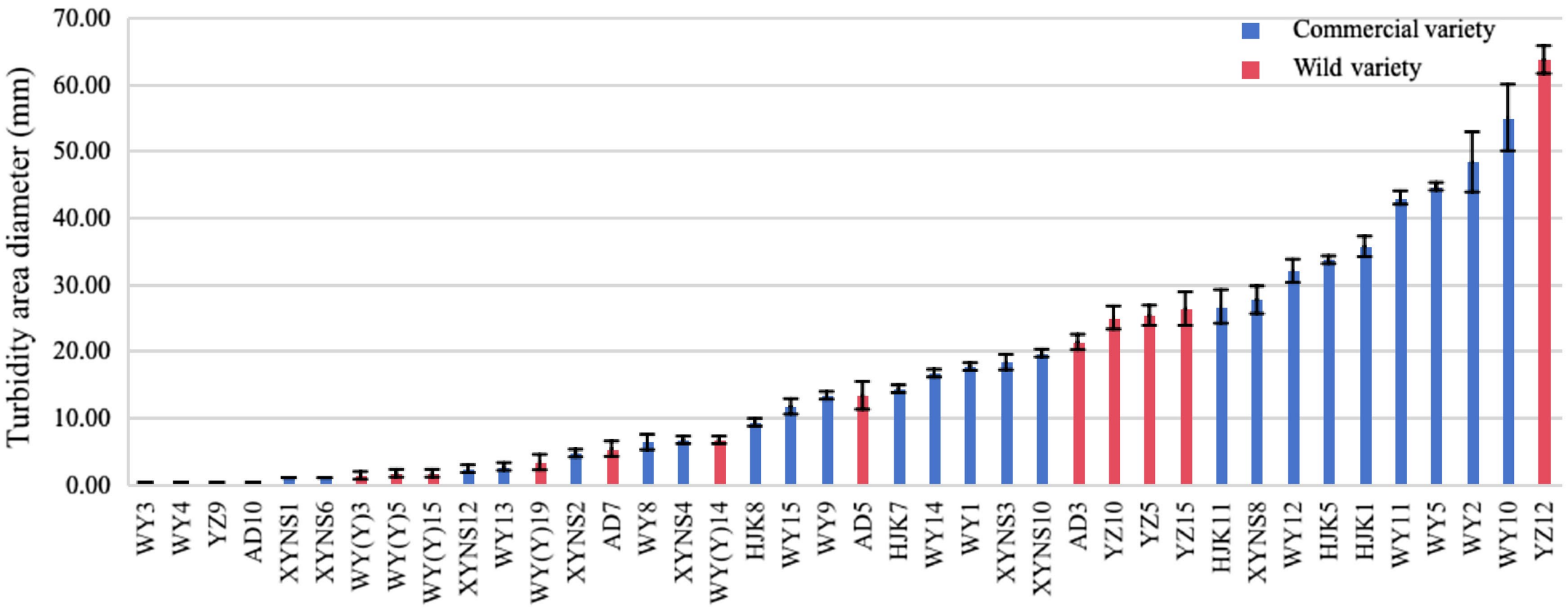
The diameter of turbid area formed by cultivable seed-borne bacteria in *Poa pratensis* L. on swimming medium.

After studying the diameter of the turbid area formed by the bacterial strains on the swimming medium, it was found that the swimming motility of different bacterial strains of bacteria in *Poa pratensis* L. showed great differences, as shown in the Figure 7. There were 10 bacterial strains with swimming diameter less than 10 mm, accounting for 76.92% % of the strains isolated from commercial varieties. There were 9 bacterial strains with swimming diameter in the range of 10–30 mm, accounting for 34.62%; there were 7 bacterial strains with swimming diameter greater than 30 mm, accounting for 26.92%. It can be seen that the distribution of the swimming ability of the isolated commercial varieties with bacteria is balanced. Among the isolated wild species seed-borne bacteria, there were 8 bacterial strains of wild species seed-borne bacteria among the bacterial strains with a swimming diameter of less than 10 mm, accounting for 57.14% of the isolated wild species seed-borne bacteria strains, and occupying the dominant position in the isolated bacterial strains. There were 5 bacterial strains with swimming diameter in the range of 10–30 mm, accounting for 35.71%; there was only 1 bacterial strain with a swimming diameter greater than 30 mm, accounting for only 7.14%, as shown in Figure 6. This shows that the swimming motility of wild species with seed-borne bacteria is weaker than that of commercial varieties with seed-borne bacteria. However, after one-way ANOVA analysis of variance, there was no significant difference in bacterial motility between commercial varieties and wild species (*p* = 0.377).

### 3.8 Analysis of relationship

**Figure 8:**
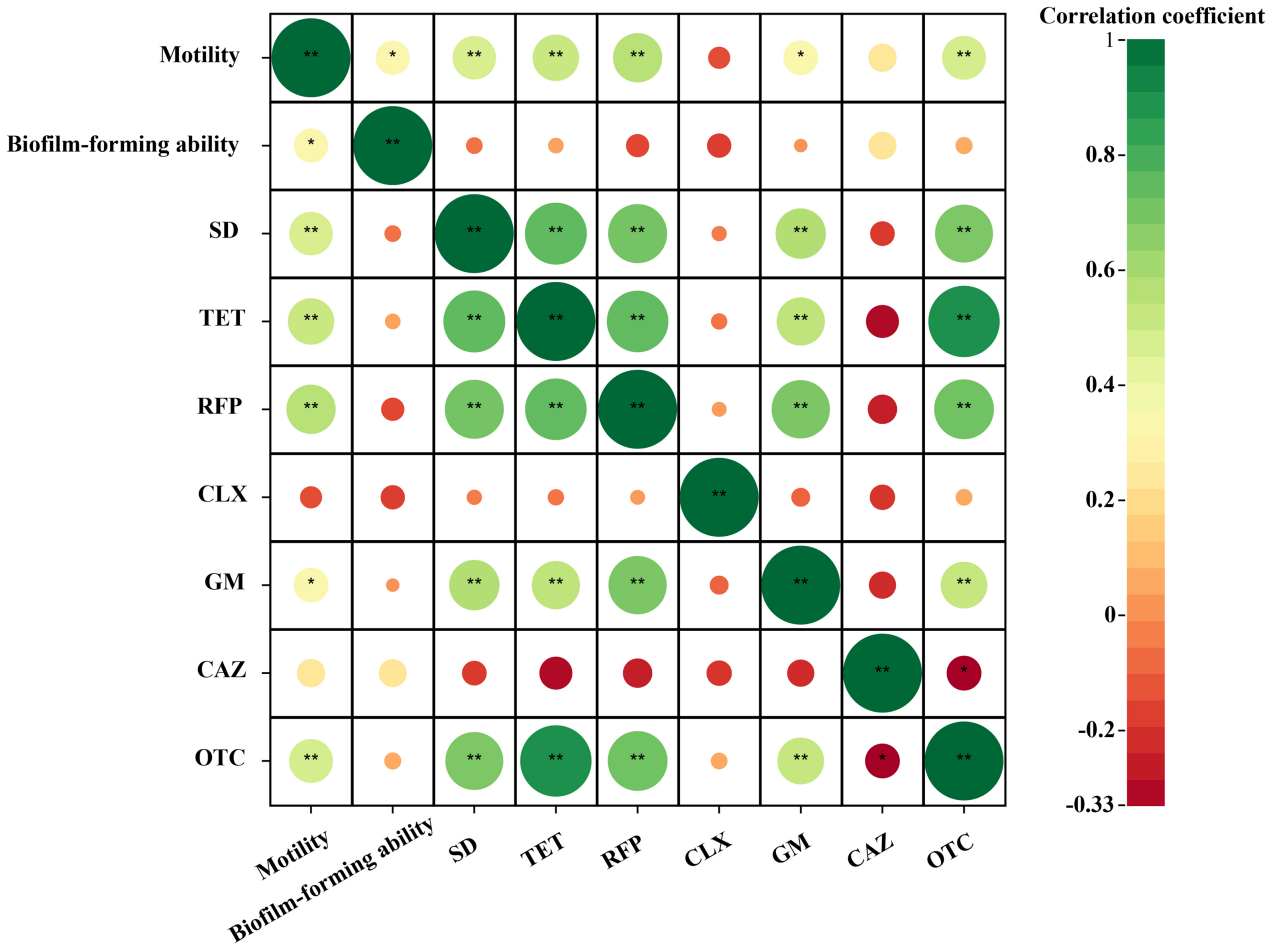
Correlation heat map of cultivable bacterial resistance and biofilm formation capability and swimming motility in *Poa pratensis* L. |*r*| < 0.3 indicates low correlation, 3 ≤ |*r*| < 0.8 indicates moderate correlation, |*r*| > 0.8 indicates high correlation. * means *p* < 0.05, ** means *p* < 0.01, there is statistical significance.

After analyzing the correlation between the resistance of the isolated bacterial strains to different types of antibiotics and their swimming motility and biofilm formation ability, it was found that among the antibiotics studied, the resistance of the isolated bacterial strains to antibiotics except ciprofloxacin was positively correlated with the swimming mobility of the bacterial strains, as shown in the Figure 8. There was a significant moderate positive correlation between the resistance of the strains to sulfadiazine, tetracycline, rifampicin and oxytetracycline and the swimming motility of the bacterial strains (3 ≤ |*r*| < 0.8, *p* < 0.01). There was a significantly low positive correlation between the resistance of the bacterial strains to gentamicin and the swimming motility of the bacterial strains (|*r*| < 0.3, *p* < 0.05). There was a positive correlation between the resistance of bacterial strains to tetracycline, gentamicin, ceftazidime and oxytetracycline and the ability of biofilm formation, but it was not significant. In addition, there was no significant negative correlation between the bacterial strains and the other three antibiotics. Through the correlation analysis between biofilm formation ability and swimming motility, it was found that there was a significant moderate positive correlation between them (3 ≤ |*r*| < 0.8, *p* < 0.05). In summary, the bacteria showed the following rules for the above antibiotics except ciprofloxacin. The stronger the swimming motility, the stronger the resistance to antibiotics. In addition, the stronger the bacterial swimming motility, the stronger the biofilm formation ability (Graphical Abstract).

## 4 Discussion

Seed is an important carrier of beneficial microorganisms and pathogens. A large number of microorganisms are colonized on the surface and inside of seeds (Truyens et al., 2015; Chimwamurombe et al., 2016). In this experiment, a total of 58 bacterial isolates were isolated. After identifying 40 representative bacterial isolates, 40 bacterial strains of bacteria were found to belong to 3 phyla, 7 families, and 11 genera. At the level of phylum classification, the isolated bacterial strains belonged to Firmicutes, Proteobacteria and Actinobacteria. This is basically consistent with the results of previous studies. Previous studies have found that in a wide range of plant groups, seed-related bacteria mainly exist in Firmicutes, Proteobacteria, Actinobacteria and Bacteroidetes. This shows that these bacteria occupy a dominant position in soil and aquatic ecosystems, so that the probability of seeds encountering these bacteria during growth and development is greater (Fierer et al., 2012; Shafi et al., 2017). After further analysis at the genus level, it was found that *Bacillus* spp. and *PaeniBacillus* spp. were dominant among the microorganisms colonized in the seeds. In particular, *Bacillus* sp. is a common genus among the six tested species of seed-borne bacteria, accounting for the highest proportion of all isolated seed-borne bacterial strains, reaching 37.5%, and the relative abundance of *Bacillus* spp. in seed-borne bacteria of wild *Poa pratensis* L. from Yuzhong reached 62.5%. This is similar to the results of Xu et al., who isolated 84 bacterial strains of bacteria from the seeds of four *Solanum lycopersicum* L. varieties from *Bacillus* spp. However, Adam et al. also studied the seed-borne microorganisms of different bacterial strains of *Cucurbita pepo* L., and found that the bacteria of Enterobacteriaceae dominated the seed-borne microorganisms (Adam et al., 2018). After analyzing the reasons why the results of this study are different from those of previous studies, the following points are drawn. First of all, different genotypes of seeds and different environmental conditions of seed and seedling development will cause differences in the composition of their microbial communities (Nelson et al., 2018). The second reason is that different research methods lead to differences in research results. The bacterial strains obtained by Xu et al. is cultivable bacteria, which is the same as this experiment. This method makes most microorganisms unable to be cultured, resulting in a large number of missing microbial genetic information. Secondly, microorganisms have certain selectivity to the medium, and the medium owned by the laboratory cannot meet the needs of all microbial growth, which has limitations. The study of Adam et al. used the method of 16S rRNA gene amplification and sequencing analysis, which can reflect the information of bacterial community more comprehensively (Adare et al., 2011). In addition, the number of bacteria isolated from *Poa pratensis* L. seeds in this study was between 10^4^–10^5^ CFU · g ^−1^, which was consistent with the results of previous studies. Studies have shown that the number of bacteria on seeds is 10^4^–10^8^ CFU · g ^−1^ (Compant et al., 2011; Hameed et al., 2015; Chimwamurombe et al., 2016).

The analysis of community composition and structure showed that there were some tissue differences in the distribution of seed-borne bacteria in the seeds of 6 samples. In contrast, “Lucky Goddess” had the highest specificity and the most complex community structure, while “Black Jack”, Anding wild *Poa pratensis* L. and Yuzhong wild *Poa pratensis* L. had lower specificity and single community structure. Compared with the seed-borne bacterial community structure of commercial varieties and wild species, the specificity of commercial varieties is higher and the community structure is more complex. In addition, the analysis found that the main phylum, dominant genera and some low-abundance bacterial populations of the species-borne bacteria in different tested seeds. Firmicutes was the dominant phylum shared by the tested seed-borne bacteria. This suggests that many taxa found in different test seeds may have similar sources. Firmicutes was the dominant phylum shared by the tested seed-borne bacteria. This suggests that many taxonomical group in different test seeds may have similar sources. Studies have confirmed that the colonization of Firmicutes on plants can protect their hosts from diseases or promote the growth of host plants, and also plays an important role in ecology (Kumar et al., 2012). *Bacillus* spp. accounted for the highest proportion at the genus level, which not only appeared in the six tested seed-borne bacteria, but also accounted for 37.5% of all isolated bacterial strains. *Bacillus* sp. is an important microbial population in soil and plant tissues, which can produce a large number of secondary metabolites. These secondary metabolites can affect the environment and plant health by causing changes in microbial communities (Borriss, 2015). In addition, *Bacillus* sp. has the potential to degrade antibiotics in the environment. Some researchers have studied *Bacillus thuringiensis* of *Bacillus* sp. and found that after 24 h of degradation of 1 μM erythromycin, the removal and degradation efficiencies were as high as 77 and 53%, respectively (Zhou et al., 2018). Subsequently, the function of the this genus and other advantage bacterium group can be further studied and analyzed, which provides theoretical support for the study of biological agents to replace the use of antibiotics, and provides theoretical support for the degradation of antibiotics.

The 40 seed-borne bacteria isolated in this experiment showed different degrees of resistance to different kinds of antibiotics. This is mainly because the physiological structure and related biological characteristics of different bacterial strains are different, which makes them produce different drug resistance mechanisms. Studies have found that bacteria can reduce the damage of antibiotics and fungicides to bacteria and enhance bacterial resistance by forming biofilms to reduce the passage of active molecules, changing the permeability of biofilms, and using motility to make cells escape from harsh environments (Pan et al., 2006; Smith and Hunter, 2008; Høiby et al., 2010; Koskella et al., 2011). In addition, this study also found that different types of antibiotics have different antibacterial abilities against the same bacteria. This is due to the different sterilization mechanisms of different antibiotics. Studies have shown that antibiotics such as penicillins, cephalosporins, carbapenems, and vancomycin kill bacteria by damaging or inhibiting the synthesis of bacterial cell walls. Other antibiotics play a role by affecting bacterial genetic material (quinolones and rifampicin), protein (aminoglycosides, chloramphenicols, tetracyclines, and macrolide antibiotics), or metabolism (trimethoprim and sulfonamides) (Rosenblatt-Farrell, 2009).

It is worth mentioning that this study found that the resistance of bacteria isolated from commercial varieties of *Poa pratensis* L. to antibiotics was stronger than that of wild species. This is mainly related to their different production areas. In the origin of commercial seeds, in order to ensure the growth of lawn and the quality of grass seeds, people will increase the rate of lawn formation, lawn coverage, grass seed yield, and biomass production through fertilizing in the production process, or reduce the occurrence of diseases and insect pests in the lawn by applying pesticides such as insecticides, fungicides and herbicides. The wild species *Poa pratensis* L. are mostly grown in natural grasslands or managed rough artificial grasslands, and the use of fertilizers and pesticides in these areas is much less than that of commercial crop producing areas. Therefore, the amount of residual antibiotics in the environment of *Poa pratensis* L. seeds in commercial varieties producing areas is greater than that in wild species growing areas. The commercial variety of *Poa pratensis* L. grown in a higher concentration of antibiotic residues has long been in an environment containing sub-inhibitory concentrations of antimicrobial substances, which has a significant effect on the physiology and evolution of their seed-borne bacteria. They can induce bacterial gene expression, so that some bacteria without “intrinsic antibiotic resistance” can produce drug resistance (Andersson and Hughes, 2014; Morita et al., 2014; Khurshid et al., 2017). Another important reason is that bacteria can obtain antibiotic resistance genes by mutation or horizontal transfer of genes to show antibiotic resistance (Choraine, 2010). In the commercial grass production base, the number of drug-resistant bacteria in the environment will increase due to the large number of residual antibiotics in the environment. The resistance genes in these drug-resistant bacteria may enter the seed-borne bacteria through horizontal gene transfer. This explains why the bacterial resistance of commercial *Poa pratensis* L. varieties is generally higher than that of wild species. This part of the study conducted a preliminary investigation on the distribution of drug-resistant bacteria in different types of *Poa pratensis* L. seeds, and provided support for the study of the spread range and transmission routes of drug-resistant bacteria or drug-resistant genes between plants and the environment.

The strength of bacterial resistance is related to a variety of biological characteristics of bacteria. For example, bacterial resistance can be produced by changing the structure of bacterial porin protein to reduce the permeability of bacterial biofilms (Ghai and Ghai, 2018), changing the binding sites of antibiotics (Witzky et al., 2019), and destroying antibiotics by hydrolases (Khameneh et al., 2019). For bacteria, they may be resistant to antibiotics and fungicides through one or more of the above mechanisms, resulting in resistance. For bacteria, they can resist antibiotics and fungicides through one or more of the above mechanisms, resulting in antibiotic resistance (Khameneh et al., 2019). This study mainly studied its biofilm formation ability and the swimming motility that can help it escape from adverse environments, and explored the correlation between bacterial biofilm formation ability and swimming motility and its drug resistance.

It was found that all bacterial strains had biofilm formation ability among all the isolated bacteria of *Poa pratensis* L. Among them, the bacterial strains with strong and medium biofilm formation ability accounted for 30% of all isolated bacterial strains, and the bacterial strains with weak biofilm formation ability accounted for 70%. Penesyan’s research shows that biofilms are a major way of life for microorganisms and play an important role in protecting microorganisms by providing them with a safer environment (Penesyan et al., 2021). This study also confirmed that biofilm formation is a common bacterial lifestyle. By comparing the bacterial biofilm formation ability of commercial varieties and wild species, it was found that the number of bacterial strains with strong and medium bacterial biofilm formation ability of commercial varieties accounted for 76.92% of the bacterial strains isolated from commercial products, while the number of bacterial strains with strong and medium bacterial biofilm formation ability of wild species accounted for 14.29%. The results showed that the biofilm formation ability of commercial varieties with bacteria was stronger than that of wild species. There are few reports on the causes of this result in China and abroad. However, in terms of the relationship between the formation of bacterial biofilm and bacterial resistance, the results may be related to the residual antibiotics in the host growth environment and the production of bacterial resistance. Nagasawa et al.’s study on the biofilm of *Streptococcus mutans* showed that the formation of biofilm was stimulated under the pressure of sub-inhibitory concentrations of antibiotics and contributed to a higher level of horizontal gene transfer (Nagasawa et al., 2020). However, there are differences in antibiotic residues in the seed growth environment of commercial varieties and wild species. Compared with wild species of seed-borne bacteria, commercial varieties of seed-borne bacteria have long been in an environment containing sub-MIC levels of antimicrobial substances, which stimulates the formation of biofilms. Therefore, the biofilm formation ability of commercial varieties with seed-borne bacteria is stronger than that of wild species.

Based on the influence of motility on the stage of adhesion in the process of bacterial biofilm formation, and the correlation between motility and bacterial drug resistance (Stabryla et al., 2021; Zheng et al., 2021), the swimming motility of the isolated strains was studied in this experiment. In this study, it was found that 90% of the bacterial strains in the seed-borne bacteria of *Poa pratensis* L. had swimming ability, but the swimming motility of different bacterial strains was quite different. This also proves that the motility of bacteria is a widespread feature. Studies have shown that the motility of bacteria can provide cells with some advantages for the smooth progress of related processes (such as colonization) with the host (Palma et al., 2022), and swimming is the most direct apparent behavior of prokaryotes. Although little is known about the mechanism of bacterial swimming behavior, a large number of studies have shown that bacterial cells with swimming motility can move to more resource-rich areas or away from harmful areas through their chemotaxis (Wadhams and Armitage, 2004; Hazelbauer et al., 2008; Bi and Sourjik, 2018). In addition, the swimming motility of bacteria also plays a very important role in biofilm formation. Gutman et al. have found that surface attachments (such as flagella) of motility bacteria can play an important role in the adhesion process by inducing motility bacteria to produce faster dynamic response to surface characteristics. Once the bacteria adhere to the host surface, motile bacteria can attract free bacteria through chemotaxis and quorum sensing, thus forming biofilms faster than non-motile bacteria (Gutman et al., 2013). The verification of this result requires further analysis of the correlation between the swimming motility of the strain and the biofilm formation ability.

Through further correlation analysis, it was found that there was a significant positive correlation between the resistance of the isolated bacterial strains to the other five antibiotics except ciprofloxacin and gentamicin and the swimming motility of the bacterial strains. This confirms the view that bacterial motility plays an important role in the formation of bacterial resistance. This is similar to the results of Stabryla et al., who found that highly motile *Escherichia coli* strains evolved resistance to AgNPs during repeated passages of AgNPs, while non-motile *E.coli* strains did not evolve resistance during repeated passages with AgNPs (Stabryla et al., 2021). Then, based on the important role of bacterial swimming motility in biofilm formation, the correlation between the two was further analyzed. This study showed that there was a significant moderate positive correlation between the swimming motility of the isolated strains and their biofilm formation ability, which confirmed the study of Gutman et al. In the study of the correlation between biofilm formation ability and bacterial resistance, although many studies have shown that there is a close relationship between the two, the bacterial strains isolated in this experiment have a positive correlation between the four antibiotics tested, but not significant. This is mainly because bacterial resistance can be produced through a variety of mechanisms, and for bacteria, they are resistant to antibiotics and fungicides through one or a combination of multiple mechanisms. In this case, the formation of biofilm is only one of the links, and it is not absolute. Therefore, in the future, in-depth research and analysis can be carried out on the basis of the drug resistance mechanism of its drug-resistant strains, and further study on the multiple ways of antibiotic resistance of drug-resistant bacteria, so as to provide theoretical support for solving the harm caused by harmful drug-resistant bacteria to animals, plants and the environment.

## 5 Conclusion and foresight

Microorganisms can affect plant production and performance by promoting plant growth or helping plants resist stress. Therefore, this study preliminarily discussed the diversity of cultivable bacteria in wild species and commercial varieties of *Poa pratensis* L. The diversity data showed that there were differences in the abundance of seed-borne bacterial communities in different species or varieties of *Poa pratensis* L. In addition, by comparing and analyzing the bacterial communities of commercial varieties with cultivable seed-borne bacteria and wild varieties with cultivable seed-borne bacteria, it was found that the seed-borne bacterial community of commercial varieties was more abundant than that of wild species, and the specificity of commercial varieties were more abundant than those of wild varieties, and the community structure was more complex. These diverse germ-borne bacteria can help plant seeds adapt to a variety of environments, allowing them to be planted in a wider area. In addition, this study also found that there were significant differences in antibiotic resistance, biofilm formation ability and swimming motility of different bacterial strains of cultivable seed-borne bacteria in *Poa pratensis* L. It is worth noting that the antibiotic resistance, swimming motility and biofilm formation ability of commercial varieties were stronger than those of wild species, which were related to the survival and adaptability of bacteria. Studies have shown that antibiotic residue pollution can cause stress on the growth and development of plants, and antibiotic residue pollution has become a worldwide environmental problem. In this context, the commercial variety *Poa pratensis* L. can be planted on a large scale around the world, which is inseparable from its interaction with seed-borne bacteria. One of the reasons is that the germ-borne bacteria have a variety of biological functions, and can continue to exist in new plants with the germination process of seeds, thus continuously affecting their growth and development process. In addition, some of the isolated species-borne bacteria may have the potential to alleviate the growth stress of plants under the environment of antibiotic residues or degrade the residual antibiotics in the environment, which will become the main content of the follow-up study of this study. Subsequently, we will study the degradation ability of the isolated bacterial strains to antibiotics, and screen out the functional strains to be inoculated on the *Poa pratensis* L. plants under antibiotic stress to study its mitigation effect on the growth of *Poa pratensis* L. under antibiotic stress. This study also elucidated the correlation between bacterial resistance, swimming motility and biofilm formation ability. Based on the correlation of the three, further research will be carried out on the pathways of bacterial resistance related to this aspect (Graphical Abstract).

## Data availability statement

The original contributions presented in the study are included in the article/Supplementary material, further inquiries can be directed to the corresponding author.

## Author contributions

JX: Conceptualization, Data curation, Formal analysis, Investigation, Methodology, Software, Writing – original draft, Writing – review & editing. JY: Writing – review & editing. SZ: Writing – review & editing. XH: Writing – review & editing. HC: Writing – review & editing. XB: Provided important wild germ plasm resources. ZZ: Project administration, Resources, Supervision, Writing – review & editing.

## Footnotes

^1^https://www.chiplot.online
