# Supplementary figures and images for: Study on seed-borne cultivable bacterial diversity and antibiotic resistance of Poa pratensis L
Source: Front Microbiol. 2024 Jan 31;15:1347760. doi: 10.3389/fmicb.2024.1347760 (PMC10864108; doi:10.3389/fmicb.2024.1347760)

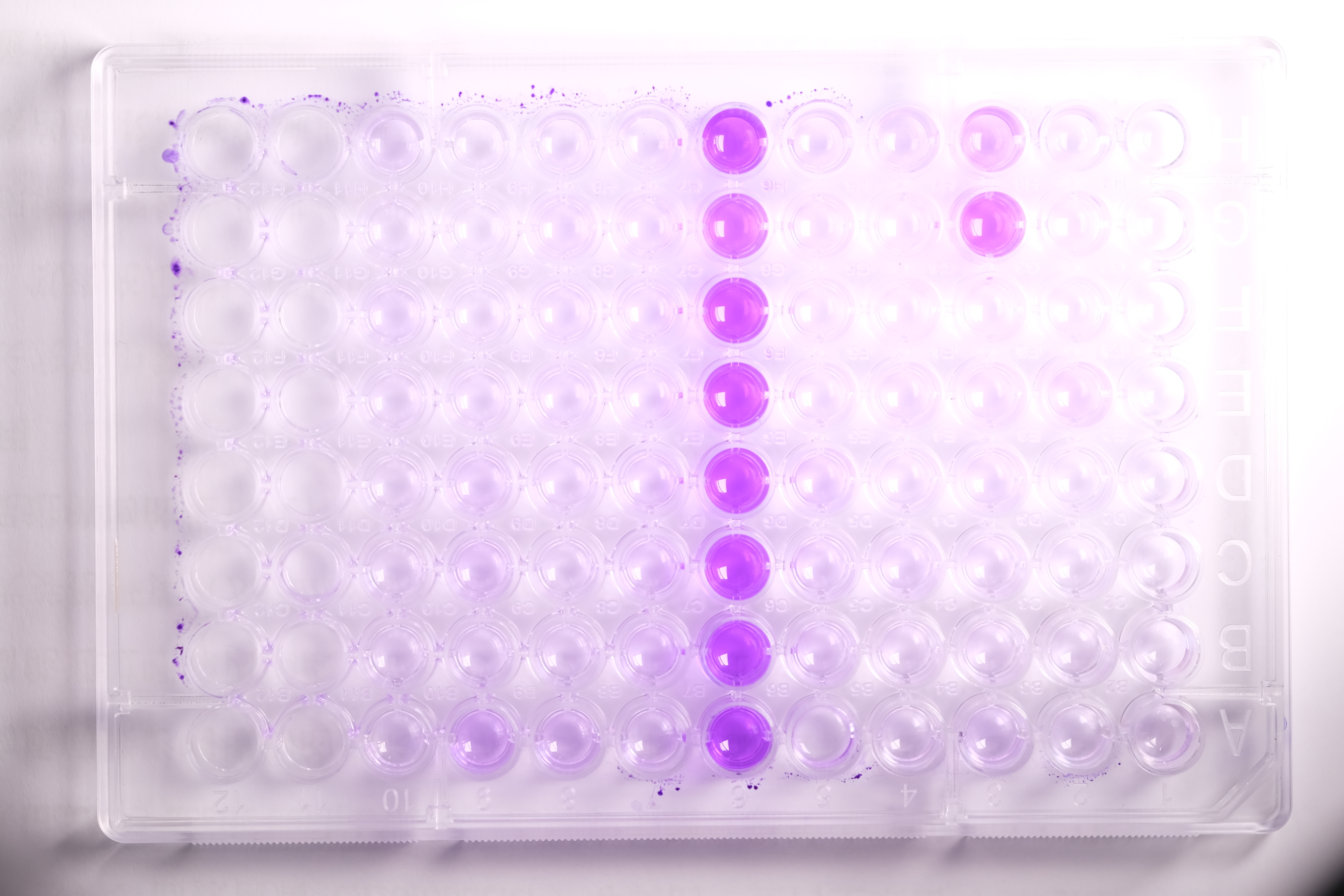

Supplement: Supplementary file 1 [file Data_Sheet_1.zip › Supplementary material/Biofilm-forming ability/Crystal violet staining.JPG]

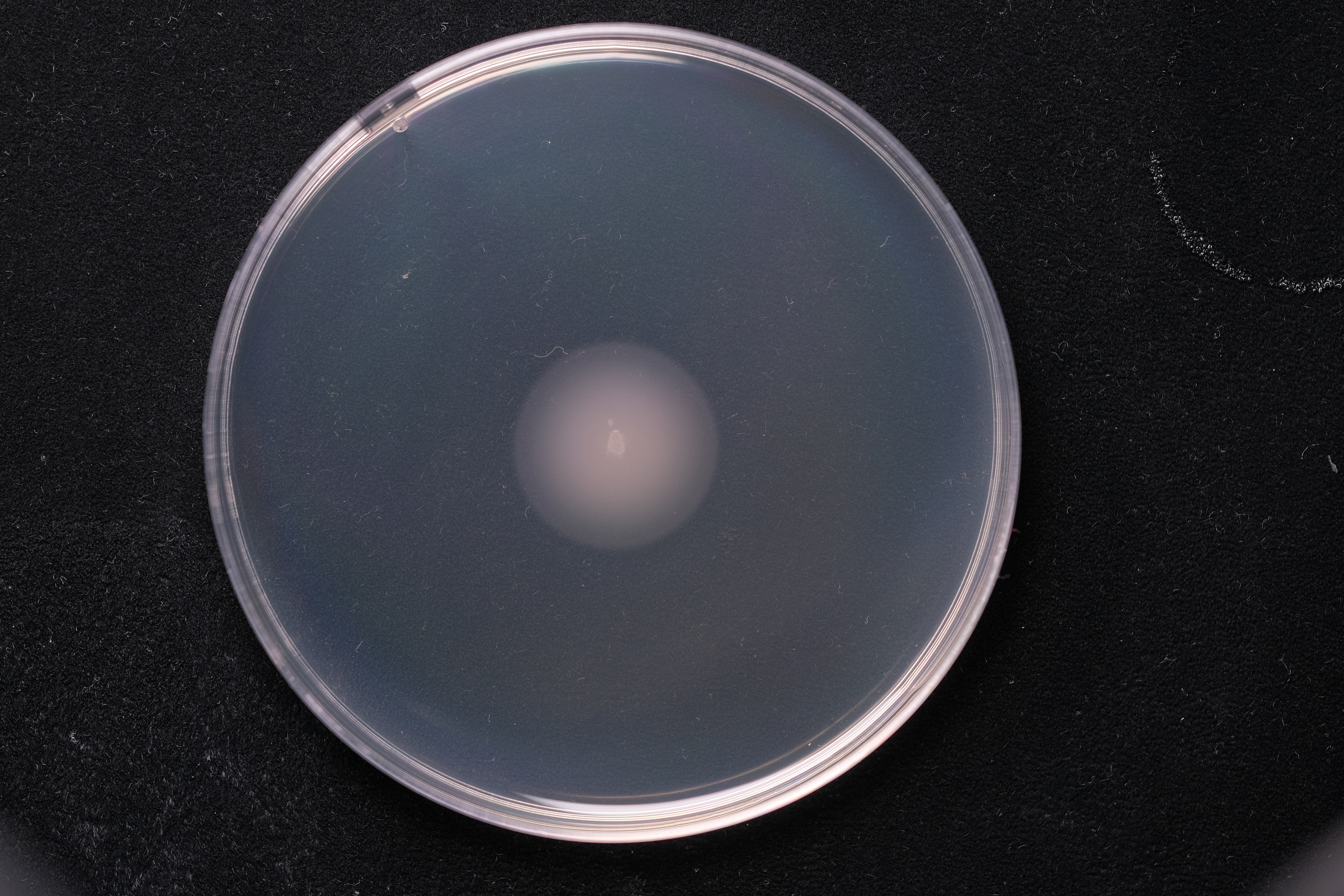

Supplement: Supplementary file 1 [file Data_Sheet_1.zip › Supplementary material/Representative pictures of swimming motility/AD3.JPG]

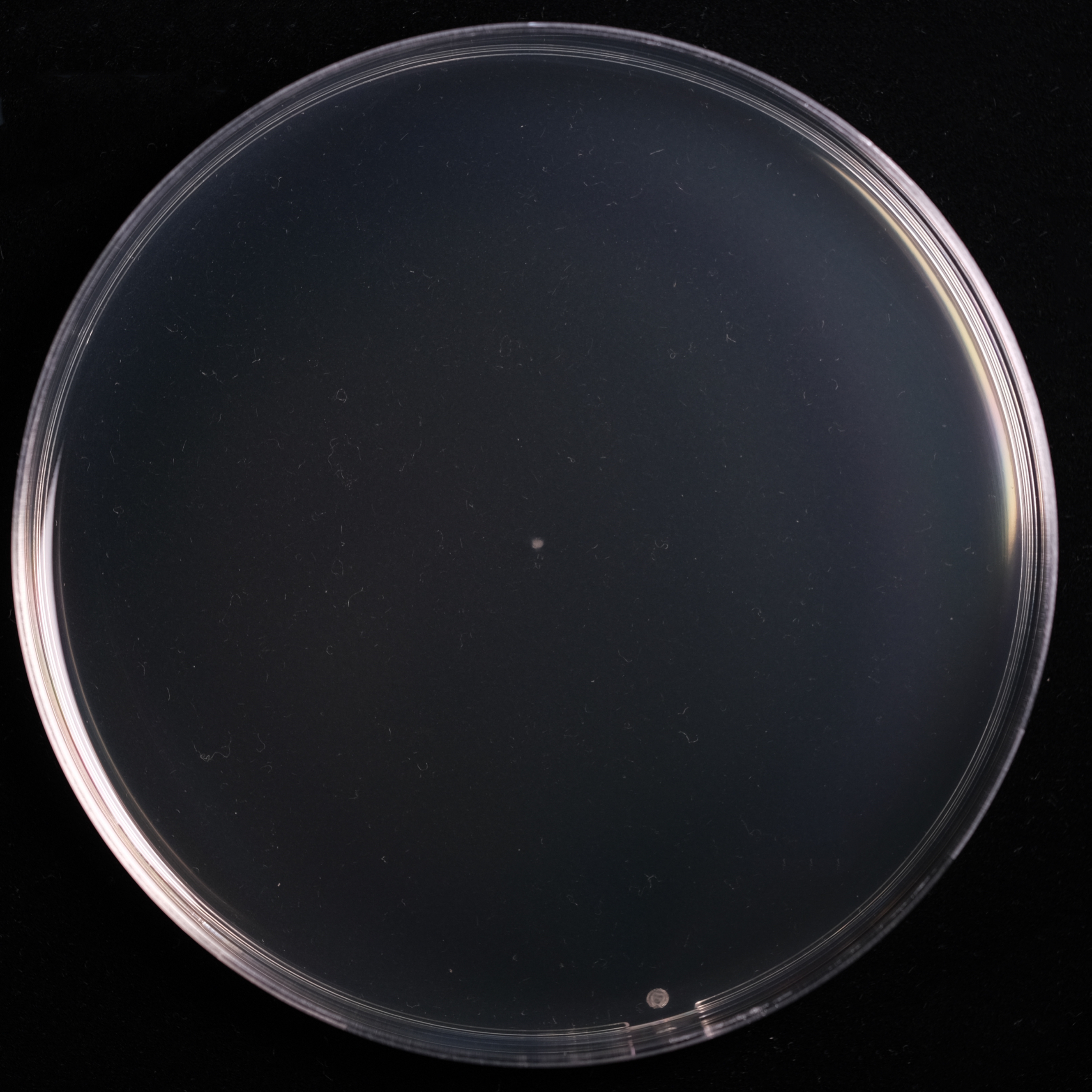

Supplement: Supplementary file 1 [file Data_Sheet_1.zip › Supplementary material/Representative pictures of swimming motility/WY3.jpg]

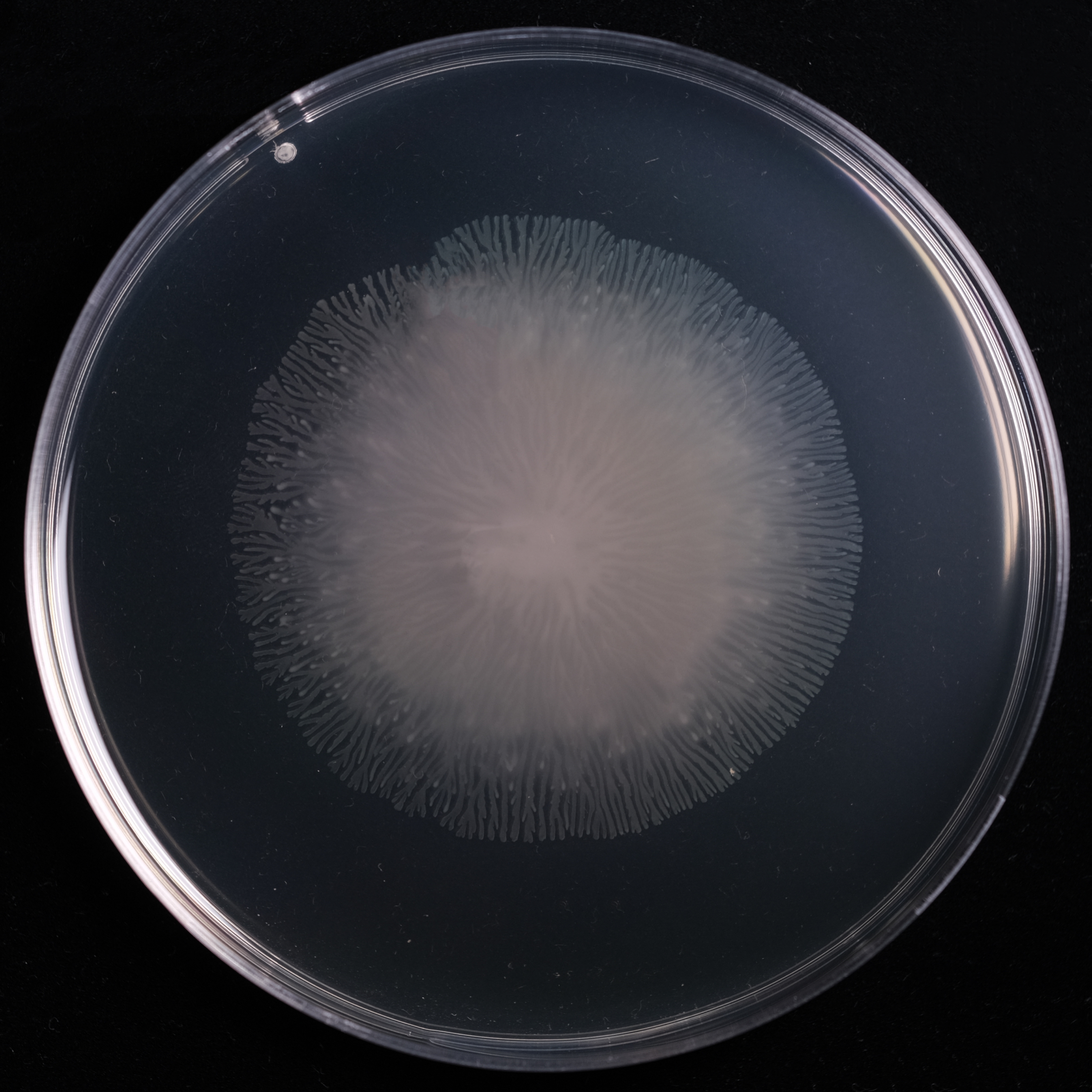

Supplement: Supplementary file 1 [file Data_Sheet_1.zip › Supplementary material/Representative pictures of swimming motility/WY2.jpg]

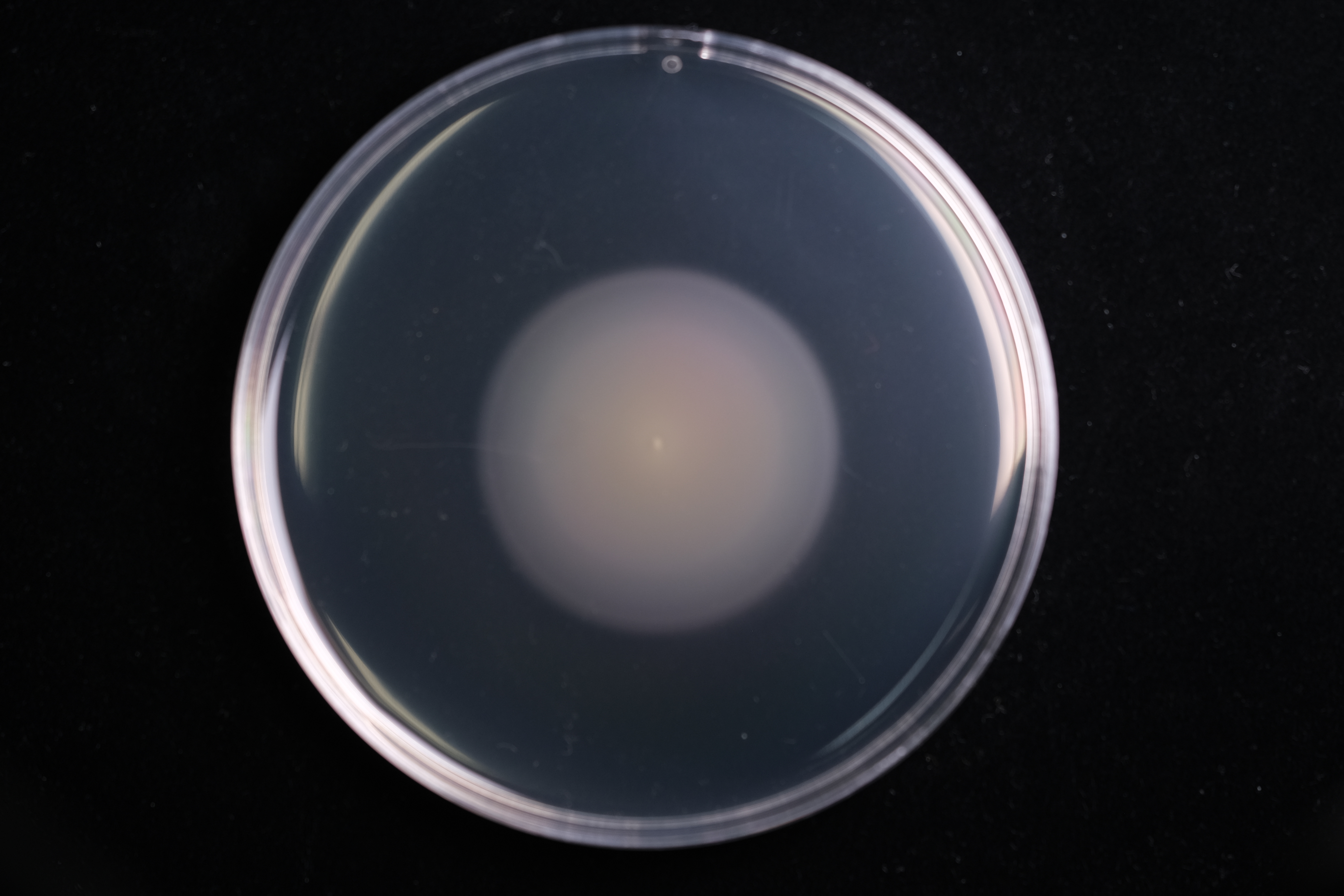

Supplement: Supplementary file 1 [file Data_Sheet_1.zip › Supplementary material/Representative pictures of swimming motility/HJK1.JPG]

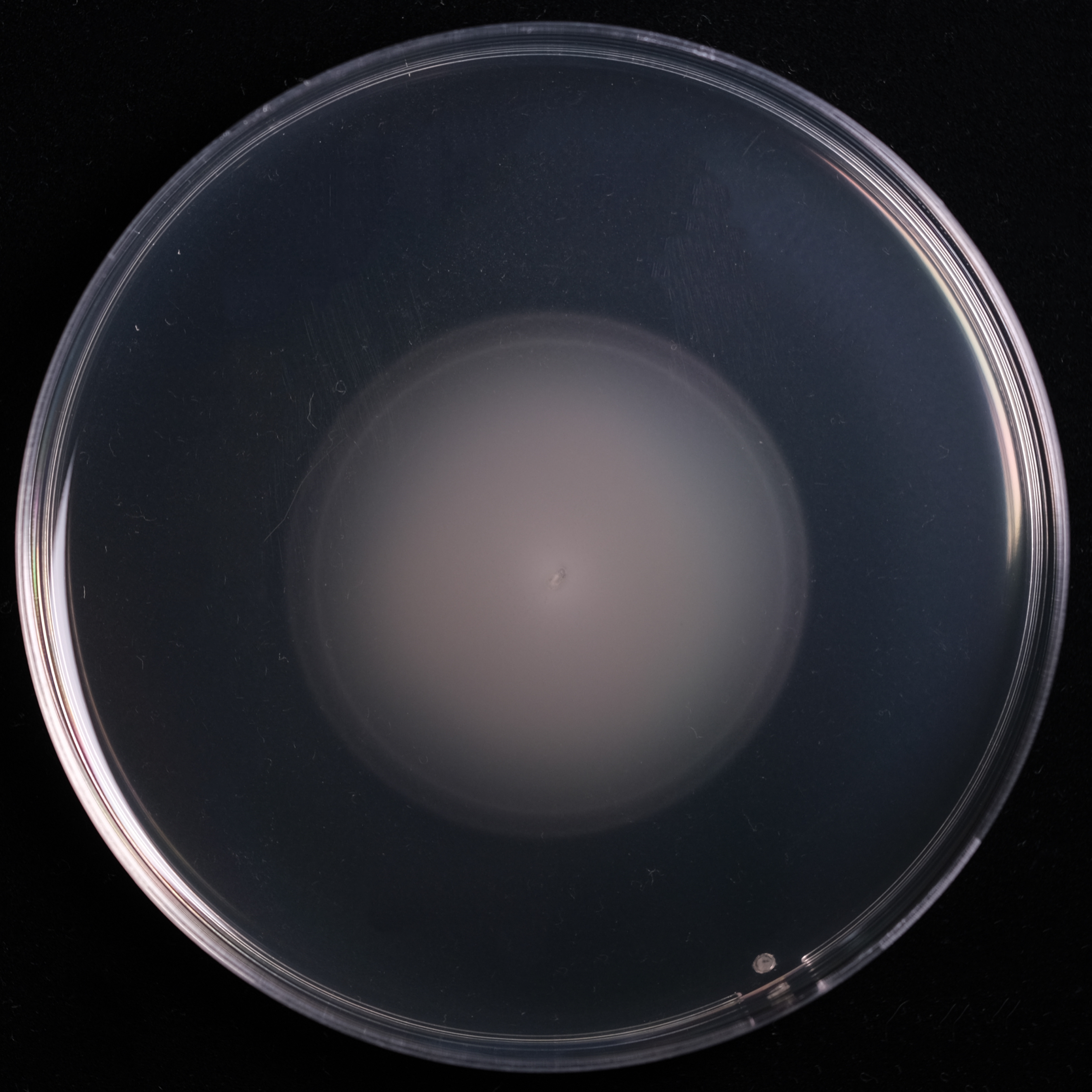

Supplement: Supplementary file 1 [file Data_Sheet_1.zip › Supplementary material/Representative pictures of swimming motility/WY11.jpg]
